# Are lipid-lowering therapies used according to the guidelines?: A real-world retrospective study of 1001 patients with type 2 diabetes

**DOI:** 10.1097/MD.0000000000047360

**Published:** 2026-01-23

**Authors:** Muhammet Fatih Şahin, Ali Erol, Gökhan Bayrak, Ali Şahin

**Affiliations:** a Department of Internal Medicine, Kestel State Hospital, Bursa, Türkiye; b Department of Internal Medicine, Bursa Yüksek İhtisas Training and Research Hospital, Bursa, Türkiye; c Department of Physiotherapy and Rehabilitation, Muş Alparslan University, Muş, Türkiye; d Department of Internal Medicine, Tokat State Hospital, Bursa, Türkiye.

**Keywords:** diabetes mellitus, diabetic neuropathy, dyslipidemias, fibrates, hydroxymethylglutaryl-CoA reductase inhibitors, hypolipidemic agents, type 2

## Abstract

Type 2 diabetes mellitus (T2DM) is frequently complicated by dyslipidemia, which increases the cardiovascular risk. Although guidelines strongly recommend lipid-lowering therapy, real-world treatment thresholds and adherence remain inconsistent in the literature. This retrospective observational study included 1001 patients with T2DM who attended outpatient clinics between March 2024 and March 2025. Demographic characteristics, metabolic parameters, and medication use (statins, fibrates, insulin, and neuropathy drugs) were retrieved from the hospital records. Group comparisons were performed using parametric and nonparametric tests, and multivariate logistic regression was used to identify the independent predictors of drug use. The mean age of the cohort was 60.9 ± 11.4 years, and 57.1% of the patients were female. The mean HbA1c was 7.47 ± 1.56%, low-density lipoprotein (LDL) was 110.7 ± 42.8 mg/dL, high-density lipoprotein was 49.6 ± 14.0 mg/dL, and triglycerides (TG) were 172.7 ± 100.1 mg/dL. Statin users had higher LDL and high-density lipoprotein levels but lower TG levels than nonusers. Fenofibrate users exhibited markedly higher TG levels. Insulin therapy was associated with poorer glycemic control but did not significantly affect the lipid parameters. In logistic regression, statin use was independently predicted by higher HbA1c and LDL levels, whereas fibrate use was predicted by elevated TG levels. Age was the only independent predictor of neuropathic drug use. In this real-world T2DM cohort, the initiation of statins and fibrates did not align with guideline-based thresholds. Statins were prescribed at higher LDL levels, whereas fibrates were reserved for patients with severely elevated TG levels. These findings highlight the persistent gap between guidelines and clinical practice and emphasize the need for strategies to improve lipid management in secondary-care settings.

## 1. Introduction

Diabetes mellitus (DM) is one of the most significant global health problems of the 21st century. According to the International Diabetes Federation Diabetes Atlas 2025, an estimated 589 million adults aged 20 to 79 years are currently living with diabetes worldwide, and this number is projected to increase to 853 million by 2050. DM imposes a substantial economic burden, accounting for approximately 9% to 10% of global health expenditures, with costs exceeding USD 760 billion annually, largely driven by type 2 DM (T2DM). This highlights the magnitude and healthcare impact of T2DM worldwide.^[1]^ Among all diabetes cases, T2DM constitutes more than 90% to 95% of all cases and remains a leading cause of morbidity and mortality through both microvascular (neuropathy, nephropathy, and retinopathy) and macrovascular complications (coronary artery disease, stroke, and peripheral artery disease).^[2,3]^

Among the metabolic abnormalities associated with T2DM, dyslipidemia is one of the most prevalent and modifiable risk factors. It is typically characterized by elevated triglyceride (TG) levels, low high-density lipoprotein (HDL) cholesterol levels, and variable low-density lipoprotein (LDL) cholesterol abnormalities, forming an atherogenic lipid profile that substantially accelerates cardiovascular disease.^[4,5]^ Recent data indicate that 60% to 70% of T2DM patients present with some form of dyslipidemia; however, despite clear evidence and guideline recommendations, optimal lipid control is often not achieved in clinical practice.^[6,7]^

Randomized controlled trials (RCTs) have consistently demonstrated that statins, which primarily lower LDL cholesterol, and fibrates, which mainly reduce TG levels, improve lipid profiles and reduce cardiovascular risk.^[8–10]^ Accordingly, international guidelines, such as those from the American Diabetes Association and the European Society of Cardiology/European Atherosclerosis Society, strongly recommend lipid-lowering therapy for patients with T2DM.^[6,7]^ However, real-world data continue to show substantial discrepancies between guideline-directed recommendations and actual practices. Therapeutic inertia, patient nonadherence, concerns about side effects, and healthcare system limitations contribute to this gap.^[11,12]^

This contradiction between controlled trial efficacy and real-world effectiveness highlights the need for further studies to explore the alignment of lipid levels, treatment patterns, and diabetic complications. The present study addresses this gap by evaluating lipid profiles, statin and fibrate use, and their association with neuropathy treatments in a large real-world cohort of more than 1000 patients with T2DM.

## 2. Materials and methods

This retrospective observational study was conducted at the Internal Medicine Clinics of Bursa Yuksek Ihtisas Training and Research Hospital and Kestel State Hospital. The study protocol was approved by the Ethics Committee of Bursa Yuksek Ihtisas Training and Research Hospital (Approval No: 2024-TBEK 2025/02-15). This study included patients who attended routine outpatient visits between March 2024 and March 2025. This study adhered to the principles outlined in the Declaration of Helsinki. Written informed consent was waived due to the retrospective design, but patient confidentiality was strictly maintained.

### 2.1. Study population

A total of 1001 patients with T2DM were retrospectively enrolled from the hospital records. All participants were Turkish adults diagnosed and followed up at Kestel State Hospital, Bursa, Türkiye. The inclusion criteria were age 18 to 90 years and a confirmed diagnosis of T2DM. Patients with type 1 diabetes, gestational diabetes, missing data, or severe comorbidities that could interfere with lipid or metabolic measurements were excluded from the study.

### 2.2. Data collection

patient information was retrospectively retrieved from electronic hospital databases and medical records of the patients. the collected data included demographic characteristics, such as age and sex, as well as metabolic parameters, including fasting plasma glucose, hba1c, and a full lipid profile consisting of hdl, ldl, tg, and total cholesterol. complete blood counts were recorded, and all biochemical parameters were assessed after at least 8 hours of fasting. in addition, information on medication use was obtained, covering oral antidiabetic drugs, insulin therapy, lipid-lowering agents (statins, fibrates, ezetimibe, and proprotein convertase subtilisin/kexin type 9 (pcsk9) inhibitors if available), and drugs prescribed for diabetic neuropathy such as α-lipoic acid, b-complex vitamins, gabapentin, pregabalin, and topical agents. medication history was verified using electronic prescriptions and detailed medical charts to ensure data accuracy.

### 2.3. Statistical analysis

All statistical analyses were performed using IBM SPSS Statistics (version 22.0, Chicago). Continuous variables were expressed as mean ± standard deviation for normally distributed data or as median with minimum and maximum values for non-normally distributed data, with distribution assessed using the Kolmogorov–Smirnov and Shapiro–Wilk tests. For group comparisons, Student *t* test was applied to normally distributed variables, whereas the Mann–Whitney *U* test was used for variables that did not follow a normal distribution. Categorical variables were analyzed using the chi-square test. Correlation analyses were performed using Pearson correlation for parametric data and Spearman correlation for nonparametric data. Furthermore, multivariate logistic regression analysis was conducted to identify the independent predictors of statin, fibrate, and neuropathy drug use. A 2-tailed *P*-value of <.05 was considered statistically significant.

## 3. Results

A total of 1001 patients with T2DM were included in this study. The mean age was 60.9 ± 11.4 years, and 57.1% of the patients were female. The mean HbA1c level was 7.47 ± 1.56%, and the mean fasting plasma glucose level was 149.9 ± 61.5 mg/dL. The lipid parameters showed a mean LDL cholesterol of 110.7 ± 42.8 mg/dL, HDL cholesterol of 49.6 ± 14.0 mg/dL, and TG of 172.7 ± 100.1 mg/dL. The baseline demographic and clinical characteristics of the study population are summarized in Table 1.

**Table 1:** Baseline characteristics of patients.

Variable	Value
Age (year)	60.9 ± 11.4
Female (n, %)	572 (57.1%)
Male (n, %)	429 (42.9%)
HbA1c (%)	7.47 ± 1.56
Fasting plasma glucose (mg/dL)	149.9 ± 61.5
HDL (mg/dL)	49.6 ± 14.0
LDL (mg/dL)	110.7 ± 42.8
Triglycerides (mg/dL)	172.7 ± 100.1

Continuous variables are presented as mean ± standard deviation (SD). Statistical significance was set at *P* <.05.

HbA1c = glycated hemoglobin, HDL = high-density lipoprotein cholesterol, LDL = low-density lipoprotein cholesterol.

When compared according to statin use, patients receiving statins had significantly higher HbA1c (7.69 ± 1.60 vs 7.38 ± 1.54%, *P* = .005), HDL (51.2 ± 13.0 vs 48.9 ± 14.4 mg/dL, *P* = .001), and LDL levels (119.2 ± 46.0 vs 107.1 ± 40.9 mg/dL, *P* <.001). Conversely, TG levels were lower in the statin users than in the nonusers (153.1 ± 68.4 vs 181.1 ± 109.8 mg/dL, *P* = .004). Among patients on fenofibrate, HDL levels were higher (54.3 ± 14.1 vs 48.9 ± 14.2 mg/dL, *P* = .001), LDL levels were lower (97.7 ± 36.8 vs 111.9 ± 42.8 mg/dL, *P* = .001), while TG were markedly elevated (315.4 ± 174.8 vs 158.1 ± 75.1 mg/dL, *P* <.001). The distribution of lipid parameters and the use of statins and fibrates among patients are presented in Table 2.

**Table 2:** Comparisons according to statin and fenofibrate use.

Variable	No statin	Statin	*P*-value	No fenofibrate	Fenofibrate	*P*-value
HbA1c (%)	7.38 ± 1.54	7.69 ± 1.60	.005	7.48 ± 1.56	7.36 ± 1.52	.472
HDL (mg/dL)	48.9 ± 14.4	51.2 ± 13.0	.001	48.9 ± 14.2	54.3 ± 14.1	**.001**
LDL (mg/dL)	107.1 ± 40.9	119.2 ± 46.0	<.001	111.9 ± 42.8	97.7 ± 36.8	**.001**
Triglycerides (mg/dL)	181.1 ± 109.8	153.1 ± 68.4	.004	158.1 ± 75.1	315.4 ± 174.8	**<.001**

Continuous variables are presented as mean ± standard deviation (SD). Bold values indicate statistically significant results (*P* < .05).

HbA1c = glycated hemoglobin, HDL = high-density lipoprotein cholesterol, LDL = low-density lipoprotein cholesterol.

In terms of insulin therapy, patients receiving insulin had significantly higher HbA1c (8.21 ± 1.67 vs 7.31 ± 1.49%, *P* <.001) and fasting plasma glucose levels (180.0 ± 81.5 vs 144.8 ± 57.0 mg/dL, *P* <.001), reflecting the fact that insulin is typically prescribed to individuals with poorer baseline glycemic control.. No significant differences were observed in the lipid parameters Comparisons of metabolic parameters according to insulin use are summarized in Table 3.

**Table 3:** Comparisons according to insulin use.

Variable	No insulin	Insulin	*P*-value
HbA1c (%)	7.31 ± 1.49	8.21 ± 1.67	**<.001**
Fasting plasma glucose (mg/dL)	144.8 ± 57.0	180.0 ± 81.5	**<.001**
HDL (mg/dL)	49.7 ± 14.1	49.3 ± 14.1	.749
LDL (mg/dL)	110.0 ± 42.9	113.6 ± 42.5	.329
Triglycerides (mg/dL)	170.3 ± 96.4	183.3 ± 114.5	.100

Continuous variables are presented as mean ± standard deviation (SD). Bold values indicate statistically significant results (*P* < .05).

HbA1c = glycated hemoglobin, HDL = high-density lipoprotein cholesterol, LDL = low-density lipoprotein cholesterol.

In the multivariate logistic regression analysis, the independent predictors of statin use were higher HbA1c (OR = 1.21, *P* <.001), higher LDL (OR = 1.006, *P* <.001), and lower TGlevels (OR = 0.996, *P* <.001). Independent predictors of fenofibrate use were higher TG (OR = 1.014, *P* <.001) and lower HbA1c (OR = 0.76, *P* = .006). For neuropathy drug use, only age was a significant predictor (OR = 1.02, *P* = .041). The results of the multivariate logistic regression analysis are shown in Table 4.

**Table 4:** Multivariate logistic regression analysis.

Dependent Variable	Independent Variable	OR	95% CI	*P*-value
Statin	HbA1c	1.21	1.08–1.35	**<.001**
	LDL	1.006	1.003–1.010	**<.001**
	Triglycerides	0.996	0.994–0.998	**<.001**
Fenofibrate	HbA1c	0.76	0.62–0.93	**.006**
	Triglycerides	1.014	1.011–1.017	**<.001**
Neuropathy drug	Age	1.02	1.00–1.04	**.041**

Bold values indicate statistically significant results (*P* < .05).

CI = confidence interval, HbA1c = glycated hemoglobin, HDL = high-density lipoprotein cholesterol, LDL = low-density lipoprotein cholesterol, OR = odds ratio.

Trend analysis revealed that the proportion of patients receiving statins increased with higher HbA1c levels (<6.5%: 24.2%, ≥8.5%: 36.1%; *P* = .010). No significant trend was observed for fenofibrate or neuropathy drug use across the HbA1c strata, whereas insulin use markedly increased with increasing HbA1c levels (<6.5%: 9.3%, ≥8.5%: 32.6%; *P* <.001).

## 4. Discussion

In this real-world cohort of 1001 patients with T2DM, we observed distinct treatment patterns for lipid-lowering and neuropathy therapies. Our findings demonstrated that statin users had significantly higher HbA1c and LDL levels but lower TG levels than nonusers. Fenofibrate users exhibited markedly higher TG levels despite lower LDL levels. Patients receiving insulin had higher HbA1c and fasting glucose levels, reflecting the fact that insulin therapy is typically prescribed to individuals with poorer baseline glycemic control rather than causing poor control itself. Age was the only independent predictor of neuropathic drug use in patients with diabetes.

### 4.1. Statin therapy

RCTs have consistently demonstrated that statins reduce LDL cholesterol and cardiovascular risk in T2DM.^[9,13,14]^ However, in our cohort, LDL levels were paradoxically higher among statin users than nonusers. However, in our cohort, LDL levels were paradoxically higher among statin users than nonusers. This outcome reflects real-world therapeutic inertia and indication bias, a phenomenon frequently observed in observational studies highlighting the suboptimal achievement of lipid goals despite guideline recommendations.^[15–17]^ This discrepancy reflects the difference between highly selected RCT populations – comprising younger, adherent patients with fewer comorbidities – and real-world cohorts, which often include older, multimorbid individuals with poorer adherence.^[6,7,18]^ ROC analysis confirmed that LDL was a statistically significant but only moderate predictor of statin initiation (AUC >0.6), highlighting that treatment decisions in secondary-care practice are still largely guided by LDL thresholds rather than overall cardiovascular risk. While international guidelines (American Diabetes Association, ESC/EAS) recommend a target of <70 mg/dL for high-risk T2DM patients,^[19,20]^ our data suggest that statins are typically initiated at much higher cutoffs (>130–150 mg/dL). Beyond therapeutic inertia, barriers such as patient reluctance due to fear of side effects, poor adherence, and physician hesitancy to prescribe statins in asymptomatic patients further contribute to the gap between RCT efficacy and real-world outcomes.^[12,21]^

### 4.2. Fenofibrate therapy

RCTs such as FIELD and ACCORD have shown that fibrates significantly reduce TG levels by 25% to 50% and may modestly improve cardiovascular outcomes in selected T2DM populations.^[10,22,23]^ In contrast, our findings revealed higher TG levels among fenofibrate users than nonusers, a paradox also observed in Asian and real-world cohorts.^[24–26]^ This apparent contradiction can be explained by indication bias: fibrates are typically prescribed only to patients with markedly elevated TG levels (>300 mg/dL), whereas RCTs enroll patients across broader TG ranges. Indeed, recent registry-based analyses demonstrated that the TG gap between fibrate users and nonusers narrows after propensity matching, supporting this explanation.^[27]^

### 4.3. Insulin use

In our cohort, patients receiving insulin had significantly higher HbA1c and fasting glucose levels than nonusers. This finding reflects real-world therapeutic inertia, where initiation of insulin therapy is often delayed until the advanced stages of T2DM, despite evidence supporting earlier initiation.^[28,29]^ The association between insulin use and higher HbA1c values should not be interpreted as a causal effect; rather, it reflects indication bias, as insulin is generally initiated in patients with more advanced or poorly controlled diabetes. Biologically, this pattern corresponds to progressive β-cell dysfunction and worsening insulin resistance with a longer disease duration.^[11]^ In addition, patient fears of hypoglycemia, concerns about weight gain, and the burden of injections, coupled with physician hesitancy, contribute to the delayed initiation of insulin therapy.^[30]^ This contrasts with landmark trials such as UKPDS and ACCORD, which demonstrated that earlier and intensive glucose-lowering strategies can reduce long-term complications.^[31,32]^

### 4.4. Neuropathy treatment

In our analysis, age emerged as the sole independent predictor of neuropathy drug use, consistent with prior evidence identifying age and diabetes duration as the strongest risk factors for diabetic peripheral neuropathy.^[33,34]^ Unlike studies linking dyslipidemia, particularly elevated TG, to neuropathy progression,^[35]^ our cross-sectional design did not reveal significant associations between lipid parameters and neuropathy treatment. This may reflect the limitations of capturing neuropathy progression without longitudinal data. Notably, patients receiving gabapentin or pregabalin had higher LDL levels than those treated with alpha-lipoic acid. This likely reflects therapeutic sequencing, as alpha-lipoic acid is typically used as a first-line treatment, while gabapentin/pregabalin is reserved for more advanced or refractory cases, suggesting that treatment choice may act as a proxy for disease severity rather than metabolic control.^[36,37]^

Clinical and policy implications. Our findings highlight a persistent gap between guidelines and practice. Decision-support systems integrated into electronic health records, such as automated LDL alerts or statin initiation reminders, have been effective in improving adherence to guideline-based thresholds.^[38]^ Structured patient education programs, including diabetes self-management education, significantly improve treatment adherence and lipid goal attainment.^[39]^ Multidisciplinary care models involving endocrinologists, internists, and diabetes educators have shown promise in optimizing both lipid and neuropathy management.^[40]^ From a policy perspective, the absence of advanced lipid-lowering agents, such as ezetimibe and PCSK9 inhibitors, in our cohort reflects reimbursement and access barriers in secondary-care hospitals. Expanding insurance coverage and reducing cost barriers are essential to ensure equitable access to modern therapies.^[41]^

Limitations. This study had several limitations. First, its cross-sectional design precludes causal inferences, particularly regarding neuropathy progression. Second, prescription bias may have influenced the results, as physicians tend to initiate statins or fibrates in patients with higher lipid levels. Third, the single-center nature of our cohort may limit its generalizability to other healthcare settings. Additionally, we were unable to account for potential confounders, such as socioeconomic status, diet, physical activity, and duration of diabetes, which may have affected both lipid levels and neuropathy outcomes.^[42]^ Nevertheless, these real-world data remain valuable for highlighting treatment inertia and practice gaps in secondary care.

Future directions and overall interpretation. Future research should explore whether earlier initiation of insulin and lipid-lowering therapies can improve real-world outcomes and evaluate strategies to overcome patient- and physician-level barriers to treatment. Longitudinal cohorts are needed to clarify the metabolic determinants of neuropathy progression. In addition, registry-based pragmatic trials incorporating decision-support alerts, reimbursement reforms, and patient engagement interventions may offer scalable solutions to bridge the gap between guidelines and practice.^[38]^ Taken together, our findings emphasize that while RCTs confirm the benefits of early lipid and glucose control, treatment is often delayed until thresholds are exceeded in real-world practice. A multifaceted strategy addressing therapeutic inertia, patient adherence, and systemic barriers is essential to align clinical practice with evidence and improve outcomes in T2DM.

## Acknowledgements

The authors would like to thank the intensive care and internal medicine staff of Kestel State Hospital for their dedicated patient care and support during the data collection. Additionally, the authors acknowledge the use of OpenAI’s ChatGPT as a digital assistant for language polishing and editing support during the manuscript preparation.

## Author contributions

**Conceptualization:** Muhammet Fatih Şahin, Ali Erol, Gökhan Bayrak, Ali Şahin.

**Data curation:** Muhammet Fatih Şahin.

**Formal analysis:** Muhammet Fatih Şahin, Gökhan Bayrak, Ali Şahin.

**Investigation:** Ali Erol.

**Methodology:** Muhammet Fatih Şahin.

**Resources:** Ali Erol.

**Software:** Muhammet Fatih Şahin, Gökhan Bayrak.

**Writing – original draft:** Muhammet Fatih Şahin, Ali Erol, Gökhan Bayrak, Ali Şahin.

**Writing – review & editing:** Muhammet Fatih Şahin, Ali Erol, Gökhan Bayrak, Ali Şahin.
